# *ALPK1* missense pathogenic variant in five families leads to ROSAH syndrome, an ocular multisystem autosomal dominant disorder

**DOI:** 10.1038/s41436-019-0476-3

**Published:** 2019-04-10

**Authors:** Lloyd B. Williams, Asif Javed, Amin Sabri, Denise J. Morgan, Chad D. Huff, John R. Grigg, Xiu Ting Heng, Alexis J. Khng, Iris H. I. M. Hollink, Margaux A. Morrison, Leah A. Owen, Katherine Anderson, Krista Kinard, Rebecca Greenlees, Danica Novacic, H. Nida Sen, Wadih M. Zein, George M. Rodgers, Albert T. Vitale, Neena B. Haider, Axel M. Hillmer, Pauline C. Ng, Shankaracharya, Anson Cheng, Linda Zheng, Mark C. Gillies, Marjon van Slegtenhorst, P. Martin van Hagen, Tom O. A. R. Missotten, Gary L. Farley, Michael Polo, James Malatack, Julie Curtin, Frank Martin, Susan Arbuckle, Stephen I. Alexander, Megan Chircop, Sonia Davila, Kathleen B. Digre, Robyn V. Jamieson, Margaret M. DeAngelis

**Affiliations:** 1 Department of Ophthalmology and Visual Sciences, John A Moran Eye Center, University of Utah School of Medicine, Salt Lake City, UT USA; 2 Genome Institute of Singapore, Singapore, Singapore; 3 Eye Genetics Research Unit, Children’s Medical Research Institute, The Children’s Hospital at Westmead, Save Sight Institute, University of Sydney, Sydney, NSW Australia; 4 Department of Epidemiology, Division of OVP, Cancer Prevention and Population Sciences, The University of Texas MD Anderson Cancer Center, Houston, TX USA; 5 Discipline of Ophthalmology, University of Sydney, Sydney, NSW Australia; 6 Department of Clinical Genetics, Erasmus MC, Rotterdam, The Netherlands; 7 Drs. Farley, Polo and Ho, Colonial Heights, VA USA; 8 National Institutes of Health, National Human Genome Research Institute, Undiagnosed Diseases Network, Bethesda, MD USA; 9 National Eye Institute, National Institutes of Health, Bethesda, MD USA; 10 Department of Hematology, Utah Health Sciences Center, Salt Lake City, UT USA; 11 Department of Ophthalmology, Schepens Eye Research Institute/Massachusetts Eye and Ear, Harvard Medical School, Boston, MA USA; 12 Department of Immunology, Erasmus MC, Rotterdam, The Netherlands; 13 The Rotterdam Eye Hospital, Rotterdam, The Netherlands; 14 Nemours/Alfred I. DuPont Hospital for Children, Wilmington, DE USA; 15 Department of Haematology, The Children’s Hospital at Westmead, Sydney, NSW Australia; 16 Department of Ophthalmology, The Children’s Hospital at Westmead, Sydney, NSW Australia; 17 Department of Pathology, The Children’s Hospital at Westmead, Sydney, NSW Australia; 18 Department of Nephrology, The Children’s Hospital at Westmead, Sydney, NSW Australia; 19 Cell Cycle Unit, Children’s Medical Research Institute, University of Sydney, Sydney, NSW Australia; 20 Disciplines of Genomic Medicine, and Child and Adolescent Health, Faculty of Medicine and Health, University of Sydney, Sydney, NSW Australia; 21 Department of Clinical Genetics, Western Sydney Genetics Program, The Children’s Hospital at Westmead, Sydney Children’s Hospitals Network, Sydney, NSW Australia; 22 Department of Pharmacotherapy, College of Pharmacy, University of Utah, Salt Lake City, UT USA; 23 Department of Population Health Sciences, University of Utah School of Medicine, Salt Lake City, UT USA; 24 Present Address: School of Biomedical Sciences, The University of Hong Kong, Hong Kong, Hong Kong

**Keywords:** retinal dystrophy, ALPK1, ROSAH syndrome, ciliogenesis, genome sequencing

## Abstract

**Purpose:**

To identify the molecular cause in five unrelated families with a distinct autosomal dominant ocular systemic disorder we called ROSAH syndrome due to clinical features of retinal dystrophy, optic nerve edema, splenomegaly, anhidrosis, and migraine headache.

**Methods:**

Independent discovery exome and genome sequencing in families 1, 2, and 3, and confirmation in families 4 and 5. Expression of wild-type messenger RNA and protein in human and mouse tissues and cell lines. Ciliary assays in fibroblasts from affected and unaffected family members.

**Results:**

We found the heterozygous missense variant in the ɑ-kinase gene, *ALPK1*, (c.710C>T, [p.Thr237Met]), segregated with disease in all five families. All patients shared the ROSAH phenotype with additional low-grade ocular inflammation, pancytopenia, recurrent infections, and mild renal impairment in some. ALPK1 was notably expressed in retina, retinal pigment epithelium, and optic nerve, with immunofluorescence indicating localization to the basal body of the connecting cilium of the photoreceptors, and presence in the sweat glands. Immunocytofluorescence revealed expression at the centrioles and spindle poles during metaphase, and at the base of the primary cilium. Affected family member fibroblasts demonstrated defective ciliogenesis.

**Conclusion:**

Heterozygosity for *ALPK1*, p.Thr237Met leads to ROSAH syndrome, an autosomal dominant ocular systemic disorder.

## INTRODUCTION

Retinal dystrophy describes progressive degeneration or abnormality of photoreceptors (rods and cones) leading to vision loss, with a worldwide prevalence of approximately 1 in 3500 (ref. ^1^). Cases may be isolated or syndromic with a number of additional organs involved. Syndromal forms include metabolic conditions such as Refsum disease (MIM 266500), storage disorders such as neuronal ceroid lipofuscinosis 3 (MIM 204200), and conditions where there are primary ciliary abnormalities including Bardet–Biedl (MIM 209900), Senior–Løken (MIM 266900), and Joubert syndromes (MIM 213300) (ref. ^2^). Each of these disorders has specific clinical and/or laboratory features that facilitate diagnosis, and are usually associated with autosomal recessive inheritance.

We previously described, in Tantravahi et al.,^3^ a family with an autosomal dominant mode of inheritance of a unique constellation of ocular and systemic features that presented to the ophthalmology and hematology clinics of the University of Utah. The features common to all three affected individuals in this family included a severe retinal dystrophy of the cones and rods, optic nerve edema, mild pancytopenia, and idiopathic massive splenomegaly. Two of the affected individuals had anhidrosis and migraine headaches, with one also experiencing episodic urticaria. Despite extensive literature search and discussion with colleagues, no other similarly affected individuals were identified at that time.

Here, we describe the clinical features of four additional unrelated affected families, allowing us to phenotypically define this new ocular systemic disorder, which we call ROSAH syndrome. ROSAH syndrome is named due to the predominant features in affected individuals including retinal dystrophy, optic nerve edema, splenomegaly, anhidrosis, and migraine headache. Utilizing different genomic technologies and analysis pipelines, we link the disease-causing variant in *ALPK1* (ɑ-kinase 1 [MIM 607347], c.710C>T, [p.Thr237Met]) to ROSAH syndrome in all five families. ALPK1 is a member of a class of atypical protein kinases and our cellular, expression, and functional studies suggest a critical role in centrosome and cilia biology. This study defines the genetic cause of ROSAH syndrome.

## MATERIALS AND METHODS

The study protocol at the University of Utah for phenotyping and analysis of patients in the Utah, Virginia, and Delaware cohorts was approved by the Institutional Review Boards of the University of Utah. The Australian patients were enrolled in a research project approved by the ethics committee of The Children’s Hospital at Westmead, Sydney Children’s Hospital Network, Sydney, Australia. The patient from the Netherlands was examined in a diagnostic setting. These projects conform to the Declaration of Helsinki, and written, informed consent was obtained from all participants and from the parents of minors.

All study subjects underwent complete ophthalmic and systemic examinations as dictated by the clinical needs of each. Unaffected subjects also received a complete exam for the purpose of phenotyping, and examination locations are detailed in Supplementary Methods: Clinical Examination Locations. For all families, whole blood was obtained and genomic DNA was extracted from peripheral lymphocytes according to previously described protocols.^4–6^

### Exome sequence analysis: family 1 (Utah)

**Fig. 1:**
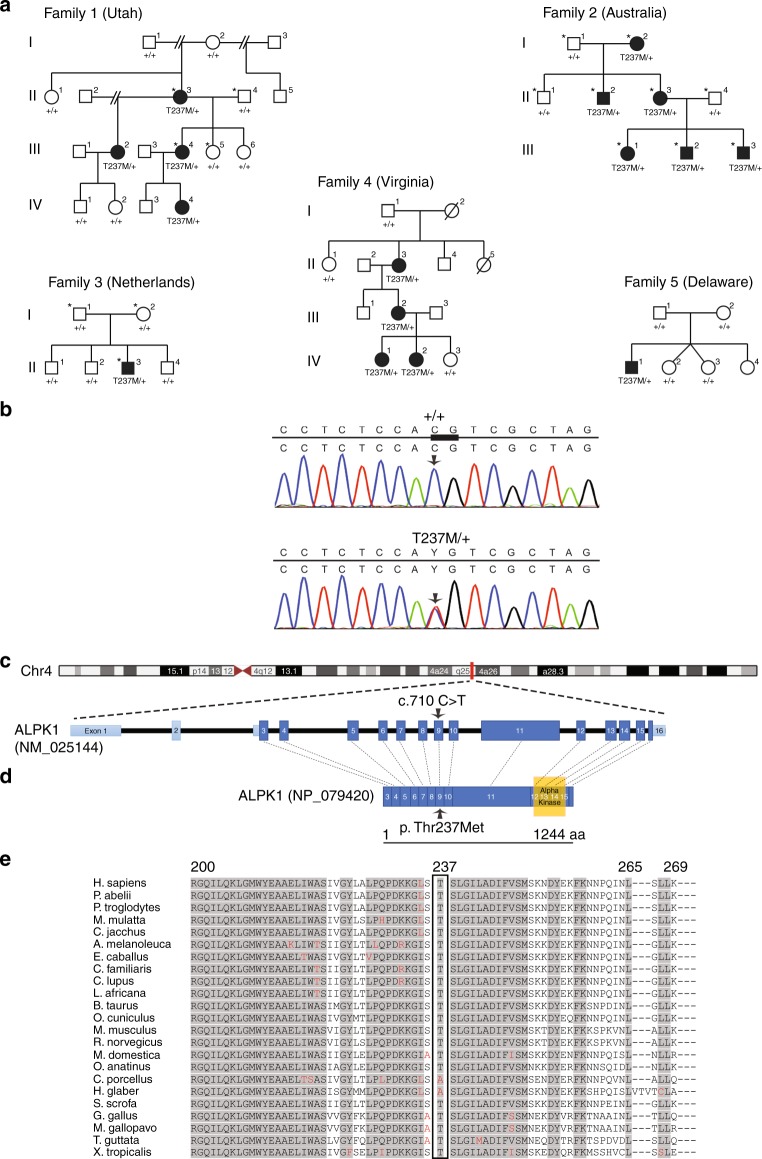
**Pedigree of families with ROSAH syndrome and pathogenic variant identification in** ***ALPK1***. (**a**) Five families affected by autosomal dominant ROSAH syndrome were analyzed in this study, from Utah (USA), Australia, The Netherlands, Virginia (USA), and Delaware (USA). Affected individuals are shaded and those examined by exome sequencing and genome sequencing in the Utah, Australia, and Netherlands families are indicated with an asterisk. Variant status is indicated below each individual for whom sequencing data was obtained (T237M indicates allele coding for [p.Thr237Met]; + indicates normal allele). (**b**) Subsequent Sanger sequencing in all families confirmed segregation of the *ALPK1* (NM_025144) variant with disease. All individuals with the variant in *ALPK1* have the ROSAH syndrome phenotype. The c.710C base pair (bp) is indicated by the arrow, and the bp 710–711 CG dimer is indicated by the thickened line. (**c**) Exonic structure of *ALPK1* (NM_025144) with the pathogenic variant on exon 9 identified in ROSAH syndrome. (**d**) Protein structure of ɑ-kinase 1 showing the location of the pathogenic variant and the location of the known kinase region. (**e**) Comparison across species for ALPK1 Thr237 and flanking sequences using multiple sequence alignment from UniProtKB/UniRef100 Release 2011_12 (14 December 2011) database. A high degree of conservation of the Thr237 amino acid in vertebrates is evident. Flanking sequences are also highly conserved. Shaded areas represent consensus sequence. Red letters represent deviation from consensus.

Exome sequencing was performed at the University of Utah on four members of the Utah cohort: the proband (III-4, in family 1, Fig. 1a), her unaffected sister (III-5), her affected mother (II-3), and her unaffected father (II-4).

After exome sequence alignment and processing (Supplementary Methods) the exome data of the family quartet was analyzed using VAAST (v2.0) (ref. ^7^) by comparing the exomes of the affected mother and daughter with a control data set consisting of 54 genomes from the Complete Genomics Diversity Panel^8^ and 1057 nonduplicative exomes from the phase I release of the 1000 Genomes Project.^9^ The analysis was restricted to the subset of variants that were present in affected mother and daughter and absent in the father and unaffected daughter. Given the extreme nature of the phenotype and rarity of the disease, all variants in our control data set and in the National Heart, Lung, and Blood Institute (NHLBI) Exome Sequencing Project (ESP) were excluded. The initial analysis resulted in a set of 44 candidate genes and none of the identified variants were located in genes known to be associated with ocular diseases such as retinitis pigmentosa or cone–rod dystrophy. Variants that were predicted to cause severe changes in protein function, were located in genes known to be expressed in retina and spleen, or were located in genes located in pathways not likely to be lethal were given highest priority. After this analysis, of 23 potential disease-causing variants, 5 were sequencing artifacts and of the others, only the *ALPK1* variant segregated appropriately with disease. The *ALPK1* variant was confirmed as a de novo pathogenic variant in the proband’s mother (family I, II-3, Fig. 1a), using the following primers: forward primer 5′-GGCAAATAGTTCATATGGAGGAA-3′ and reverse primer 5′-CCTCCCAATCTCCTGAGAAA-3′.

### Genome sequence analysis: family 2 (Australia)

Independent genome sequencing was performed for all nine members of the Australian cohort (family 2, Fig. 1a). After genome sequence alignment and processing (Supplementary Methods) a variant call list was generated. Phen-Gen^10^ was used for post–variant call analysis. Phen-Gen incorporates disease symptoms to use the phenotype to genotype link to improve the likelihood of novel disease gene identification. Phen-Gen discards common variants (minor allele frequency [MAF] >1% in dbSNP, 1000 Genomes, or ESP) and estimates the deleterious impact of pathogenic variants, allowing for a direct comparison of putative damaging variants across different classes (nonsynonymous, splice site, and indels). It further prioritizes the candidate gene list based on patient-specific symptoms by integrating available disease knowledge of phenotypically similar disorders, the genes implicated for them, and other functionally similar genes. Symptoms listed in Table 1 were used in the analysis. There were two candidate variants suggested by the analysis, but one was of low genotype quality and did not validate with Sanger sequencing. Mutation validation of the *ALPK1* pathogenic variant and segregation with disease was shown by Sanger sequencing in all family members (Macrogen Inc., Seoul, Korea) with the following primers for *ALPK1* (NM_001102406): forward 5′-TGATCCTTCAAGCTCTGTGGC-3′, and reverse 5′-CCACCAAGGACACGATTGACA-3′.

**Table 1:** ROSAH syndrome: ophthalmic and systemic features and ages of onset in affected individuals with ALPK1, p.Thr237Met, heterozyogous

	Vision impairment/optic nerve edema, age of detection (years)	Retinal dystrophy, age of assessment (years)	Splenomegaly/ pancytopenia, age of detection (years)	Anhidrosis	Headache	Renal impairment	Hepatic impairment	Other
Family 1 (Utah, USA)^3^								
II.3	10	Cone–rod, 11	15	N/K	N/K	No	No	Polyarthralgia
III.2	4	Cone–rod, 7	9	Yes	Yes, migraine	N/K	N/K	
III.4	8	Cone–rod, 13	12	Yes	Yes, migraine	No	No	
IV.4	No^a^	No^a^	1.3^b^	Yes	No^a^	Yes^c^	Yes^b^	
Family 2 (Australia)								
I.2	8	End-stage, 65	N/K	Yes	Yes, occasional	Mild	N/K	
II.2	8	End-stage, 35	18	Yes	Yes, occasional	N/K	N/K	
II.3	5	End-stage, 38	37	Yes	Yes, migraine	Mild	N/K	Episodic fevers
III.1	12	Cone–rod, 12	13	Yes	Yes, migraine	Mild	No	
III.2	7	Cone–rod, 7	No	Yes	Yes, migraine	Mild	No	
III.3	7	Cone–rod, 7	8	Yes	Yes, migraine	Mild	No	
Family 3 (Netherlands)								
II.3	7	End-stage, 26	No	Yes	Yes, occasional	No	No	Broadening of joints with stiffness, early-onset halluces valgi
Family 4 (Virginia, USA)								
II.3	Yes	34	11	Yes	Yes	No	No	Dental problem
III.2	Yes	7	14	Yes	Yes	No	No	Dental problem
IV.1	Yes	9	15 months	Yes	No	No	No	
Family 5 (Delaware, USA)								
II.1	10	Rod–cone, 13	8	N/K	N/K	N/K	Mild	Episodic fevers, back pain

*N/K* Not known.

^a^Patient only age 2 years at assessment.

^b^ɑ-1 antitrypsin deficiency present.

^c^Following *Clostridium difficile* infection and metabolic acidosis.

### Exome sequence analysis: family 3 (Netherlands)

Diagnostic exome sequencing was performed in the proband and his parents in the Netherlands cohort. After exome sequence alignment and processing (Supplementary Methods) variants were filtered with the Cartagenia software package on quality (read depth ≥10), MAF (≥0.1% in 200 alleles in dbSNP, ESP6500, 1000 Genomes Project, GoNL, or ExAC), location (within an exon or first/last 3 bp of introns). Subsequently variants were further selected based on three inheritance models (de novo autosomal dominant, autosomal recessive, and X-linked recessive). Only variants predicted to change the protein sequence (nonsynonymous single-nucleotide variants and short insertions and deletions) or in the recognition of consensus RNA splice sites were considered. Ninety-six percent of the RefSeq sequences were covered at least 10×. Confirmation of the *ALPK1* variant was performed by Sanger sequencing as described for the Utah family.

### Sanger sequencing: families 4 and 5 (Virginia and Delaware)

Identification of the *ALPK1* variant was performed in the Virginia and Delaware families at the University of Utah by Sanger sequencing using the primers as described for the Utah family.

### Alpk1 expression in the mouse

Mouse studies were approved by the Animal Care and Ethics Committee of the Children’s Medical Research Institute, Sydney, Australia. *Alpk1* (NM_027808.1) expression was examined in ARC(S) mouse female adult tissue and postnatal (P) mouse retina. RNA was extracted from samples collected from P5, P10, and adult tissues using RNeasy Lipid Tissue Mini Kit (Qiagen, Australia). Complementary DNA (cDNA) synthesis was performed using SuperScript III First-Strand Synthesis SuperMix kit (Life Technologies, Australia) according to manufacturer’s instructions. Reverse transcription polymerase chain reaction (RT-PCR) was performed using Biomix Red (Bioline, Australia) using the following primers: forward 5′-GCAAGTGCTGGATCGTCTCT-3′ and reverse 5′-TTGTTTGTATTGCCACTTTTCAGG-3′.

### ALPK1 expression in human tissues

*ALPK1* messenger RNA (mRNA) expression was examined via quantitative real-time PCR in human donor eye tissue as well as in formalin-fixed, paraffin embedded (FFPE) spleen tissue from two affected individuals from the Utah family (family 1, II-3 and III-2) along with two age-matched controls. Total RNA was extracted from macula retina and retinal pigment epithelium (RPE)/choroid, extramacula retina and RPE/choroid, and optic nerve from two normal human donor eyes using the Qiagen All-Prep Kit (Qiagen, USA). The resulting RNA was pooled for each tissue type. Total RNA was isolated from archived FFPE spleen tissue using the Qiagen All-Prep DNA/RNA FFPE kit (Qiagen, USA). cDNA was synthesized using the iScript cDNA Synthesis Kit (Bio-Rad, USA) following the manufacturer’s protocol. Real-time PCR was performed using a predesigned *ALPK1* PrimePCR gene expression assay (Bio-Rad, USA). *ALPK1* expression data was normalized to the geometric mean of three endogenous control genes (HSP90AB1, PPIA, and PSMC4), which were experimentally validated to be stably expressed in the tissue types evaluated. The resulting normalized expression data was analyzed using the comparative Ct (∆∆Ct) method.

### Cell culture and immunocytofluorescence

Primary human fibroblasts were established from the skin biopsy of affected and unaffected family members from the Australian family (Fig. 1a, family 2), and cultured in Dulbecco's Modified Eagle Medium (DMEM, Gibco) with 10% fetal calf serum, and 2-mercaptoethanol. ARPE19 cells were maintained in DMEM with 10% fetal calf serum. To enhance visualization of the primary cilium, serum starvation for 24 hours was undertaken.^11^ For centrosome and cilia analysis, human fibroblasts and ARPE19 cells were fixed in methanol in −20°C. HeLa cells were maintained in Roswell Park Memorial Institute medium (RPMI, Gibco) with 10% fetal calf serum. For multinucleation analysis, HeLa cells were transfected with *GFP-ALPK1* (WT), *GFP-ALPK1* (c.710C>T, [p.Thr237Met]), or *GFP*-only vector (empty vector) using Lipofectamine 2000 (Thermo Fisher Scientific, cat #11668019), and untransfected HeLa cells were also used as a control. The cells were fixed with 4% paraformaldehyde for 10 minutes 48 hours post-transfection. For centrosome, cilia, and multinucleation studies, after washing the coverslips with PBT (0.1% Tween 20 in phosphate-buffered saline) cells were blocked in CAS-Block (Life Technologies, Australia, cat #008120) and incubated at 4 °C overnight with primary antibodies (Supplementary Table S1). Cells were incubated with appropriate secondary antibodies (Supplementary Table S1) for 2 hours at room temperature. Images were captured using a Zeiss LSM 880 laser scanning microscope with Airyscan. Super-resolution images were captured using 63×/1.4 Oil Plan-Apochromat objective and processed using Zeiss ZEN Black software. For ciliation and multinucleation studies, data were obtained from three independent replicates where 100 cells were examined for each sample in each experiment. Statistical analysis of the results was performed using one-way analysis of variance (ANOVA) (GraphPad Prism software) and *p* values were calculated by the two-tailed *t* test.

### Immunohistofluorescence

Adult female ARC(S) mouse eyes were collected, and the corneas were removed. The eyes were embedded in optimal cutting temperature compound (Tissue-Tek, cat #4583) and snap frozen in liquid nitrogen. The paws were collected and fixed in 4% paraformaldehyde for 1 hour. Samples were then placed in 20% sucrose followed by 30% sucrose and embedded in optimal cutting temperature compound. Tissue samples were sectioned at a thickness of 14 µm. The tissue sections were blocked in a buffer consisting of 0.1% fish gelatin (Sigma Aldrich, cat #G7765), and 0.02% Triton X-100 in phosphate-buffered saline for 1 hour. Tissue sections were incubated with primary antibodies (Supplementary Table S1) overnight at 4 ºC, washed, and incubated with appropriate secondary antibodies for 2 hours. Images were captured using a Zeiss LSM 880 confocal microscope.

## RESULTS

### Clinical phenotype of ROSAH syndrome

**Fig. 2:**
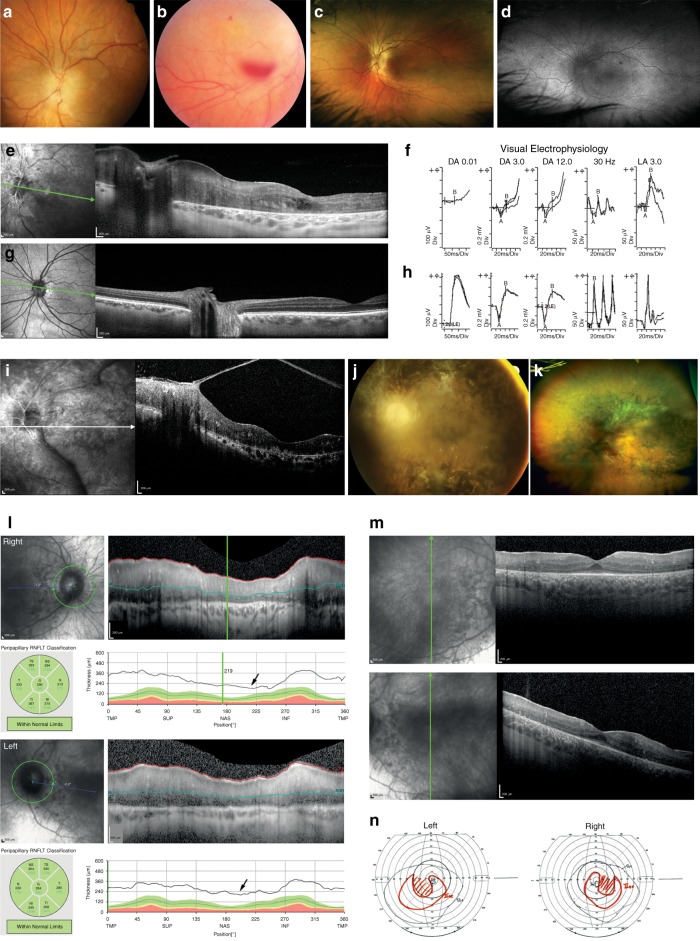
Clinical images for patients III.2 and II.3 from the Australian cohort and II.1 from the Delaware cohort.(**a**–**d**) Fundus imaging for patient III.2, Australian cohort. (**a**) Swollen optic disc with swelling extending onto the peripapillary nerve fiber layer. (**b**) Fundus image showing the vitreous and preretinal hemorrhage. (**c**) Ultrawide field fundus images color corrected with Optos viewing software (Optos plc, Dunfermline, Scotland, UK) shows prominence of the nerve fiber layer, early retinal atrophy particularly just anterior to the vascular arcades. No pigmentary changes are seen. (**d**) Ultrawide field fundus autofluoresence (FAF) (Optos plc, Dunfermline, Scotland, UK) showing diffuse hyperautofluoresence in a broad ring extending anterior to the vascular arcades and loss of the normal foveal hypoautofluorescence. (**e**, **g**) OCT. (**e**) Patient III.2 from Australian cohort shows retinal and peripapillary generalized thickening with indistinct retinal layers. The macula shows cystic changes and loss of the external limiting membrane (ELM) and photoreceptor inner segment/outer segment (IS-OS) junction. (**g**) Age-matched normal OCT is shown for comparison. (**f**, **h**) Visual electrophysiology. (**f**) Patient III.2 from the Australian cohort shows moderate photoreceptor dysfunction involving both the rod and cone systems. The DA 3.0, 30-Hz flicker, and LA 3.0 have b-wave amplitudes reduced to 50% of normal. The DA0.01 has blink artifact affecting interpretation. The pattern ERG (assessing macular function, not shown) was extinguished for the 15° field responses. The combination of electrophysiology results, OCT loss of ellipsoid zone, and reduced visual acuity led to classification as a cone–rod pattern of dysfunction. (**h**) Normal age-matched ISCEV standard ERG recordings are shown for comparison. These illustrate normal amplitudes and latencies for the scotopic (rod dependent system) DA 0.01, DA 3.0, and DA 12.0, and photopic (cone dependent system) 30-Hz flicker and LA 3.0. (**i**–**k**) OCT and fundus imaging for patient II.3, Australian cohort. (**i**) OCT shows thickening of the retina with loss of the normal retinal layer reflectance pattern and vitreous condensation (band) attaches to an epiretinal membrane. (**j**) The standard 50° fundal retinal image shows optic atrophy, marked retinal vascular attenuation, and pigmentary retinopathy. There is significant vitreous condensation overlying the retina obscuring some detail. (**k**) Ultrawide field image highlighting the extent of the pigmentary changes, which extend to the equator. (**l**) OCT retinal nerve fiber layer (RNFL), patient II.1 from the Delaware cohort, showing optic nerve edema and thickening of the peripapillary retinal nerve fiber layer (arrows). (**m**) Macula OCT, patient II.1 from the Delaware cohort, showing thickening of retina and reduction of the retinal lamination reflectance layers. (**n**) Goldmann visual fields (left and right), patient II.1 from the Delaware cohort, showing constriction of visual fields. OCT, Optical coherence tomography; ERG, Electroretinogram; ISCEV, International Society for Clinical Electrophysiology of Vision; DA, Dark adapted; LA, Light adapted; Hz, Hertz; ms, Millisecond; Div, division; TMP, Temporal; SUP, Superior; NAS, Nasal; INF, Inferior; RNFLT, Retinal nerve fiber layer thickness.

There is marked similarity in the clinical features we characterized in affected individuals from all five of these unrelated families (Fig. 1a, Table 1, Fig. 2). We propose the name ROSAH syndrome (retinopathy, optic nerve edema, splenomegaly, anhidrosis, and migraine headache), due to the consistency of the ophthalmic, hematologic, and systemic signs, and detailed clinical case reports are provided in the Supplementary Clinical Information Appendix. The most frequent earliest feature is decreased vision associated with presence of optic nerve edema on ophthalmic examination, evident as early as 4 years of age and present by age 12 years in all of the cases identified so far (Tantravahi et al.,^3^ Table 1, Fig. 2a, l). This is not associated with elevated intracranial pressure. Low-grade ocular inflammation is common in affected individuals, and does not resolve with steroid or other forms of immunosuppression. Later in childhood or the second decade of life, patients have increasing visual impairment and most have abnormal cone function and later loss of rod function (Fig. 2f). By the third decade of life, visual dysfunction is severe with visual acuity ranging from counting fingers to no light perception. In addition to the ocular findings, patients generally have splenomegaly that may begin in childhood or the second decade, and may eventually require splenectomy. Hematological assessment shows chronic mild pancytopenia, which may be exacerbated during periods of viral or other infection. Peripheral smears frequently show leukopenia with reactive lymphocytosis. Bone marrow examination is normocellular with trilineage hematopoiesis and relative erythroid hyperplasia. Affected individuals also have anhidrosis. Headaches and migraine are a frequent feature, often associated with episodic fever and back pain. Mild renal impairment was present in some members of family 2 (Fig. 1a), but is not a consistent feature in all patients.

### Heterozygous *ALPK1* pathogenic variant identified in individuals with ROSAH syndrome

Independent exome sequencing in four members of the Utah cohort (family 1, Fig. 1a), three members of the Netherlands family (family 3, Fig. 1a), and genome sequencing in all affected and unaffected family members of the Australian cohort (family 2, Fig. 1a) was conducted. Comparison of variants, also conducted independently, between affected and unaffected individuals led to the identification of a missense change in *ALPK1*, (Chr. 4q25, c.710C>T, [p.Thr237Met]) (Fig. 1b–d) as the likely disease-causing variant in each of the families. This variant arose de novo in the Utah (family 1) and Netherlands (family 3) families, and comparison of available haplotype data between the Utah and Australian (family 2) families was not consistent with a recent shared common ancestor for these two families. Two additional unrelated families with ROSAH syndrome were identified (family 4 [Virginia] and family 5 [Delaware]), and affected members were found to be heterozygous for the *ALPK1*, (c.710C>T, [p.Thr237Met]) variant, which arose de novo in the Delaware patient. All families were of non-Hispanic, Caucasian ancestry. This same C to T transition in all five unrelated families, affected the bp 710–711 CG dimer (Fig. 1b) and may represent a hotspot for mutation due to uncorrected spontaneous deamination of a methylcytosine to thymine, which is a known source of mutation in humans.^12^

Bioinformatic analysis with PolyPhen-2 (ref. ^13^) (HumVar score 0.953) predicted the *ALPK1*, (c.710C>T, [p.Thr237Met]) variant to be probably damaging, and SIFT^14^ predicted it to be damaging. The variant was highly conserved across species, with a PhastCons^15^ score of 0.986 (Fig. 1e), and was absent from the population database gnomAD. The Thr237 amino acid is in a region of poorly defined function in the protein, N terminal to the ɑ-kinase domain (Fig. 1d).

### *ALPK1* expression in the RPE, photoreceptors, and other tissues

**Fig. 3:**
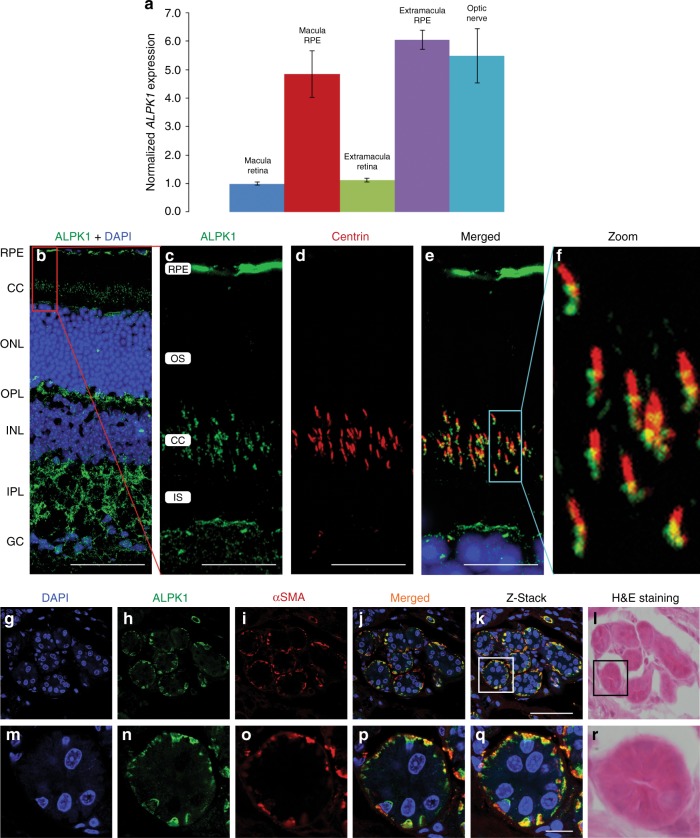
***ALPK1*** **expression in pooled normal human ocular tissues, mouse retina, and mouse sweat glands.** (**a**) Quantitative real-time polymerase chain reaction (PCR) data, normalized to the geometric mean of HSP90AB1, PPIA, and PSMC4, shows increased expression of *ALPK1* in the RPE and optic nerve tissue relative to macula retina tissue. Error bars represent the standard deviation of expression in technical replicates. (**b**–**f**) Alpk1 expression in the retinal pigmented epithelium and photoreceptor cells. (**b**) Adult mouse retinal section stained with anti-Alpk1 (green, MyBioSource, cat #MBS001969), and DAPI (blue) shows expression of Alpk1 in the RPE and connecting cilium region of the photoreceptor cells. (**c**–**e**) Magnification of the RPE and photoreceptor layers. Centrin (red) is a pancentrin antibody that marks the connecting cilium, basal body, and adjacent centriole of the photoreceptor. The merged image shows the localization of Alpk1 to the basal body region of the photoreceptor cilium and the region of the adjacent centriole. (**f**) Magnification of the connecting cilium region. (**g**–**r**) Expression of Alpk1 in mouse sweat glands: immunostaining of mouse paw sweat glands with (**g**) DAPI labeling the nuclei of cells in the skin of the mouse paw; (**h**) anti-Alpk1 antibody (green, Proteintech, cat #19107-1-AP); (**i**) anti-ɑ smooth muscle actin (α-SMA, red) as a myoepithelial cell marker. (**j**) Merged image of three channels indicates the high expression of Alpk1 in myoepithelial cells of sweat glands. (**k**) Stacked image of multiple layers of the sweat gland with 0.5-μm Z-level using confocal microscope. (**l**) Mouse sweat gland histology illustrating the morphology and location. (**m**–**r**) Magnified regions of the sweat glands encompassed by the box in (**k**) and (**l**). Scale bars represent 50 μm in (**b**) and (**k**), and 10 μm in (**c**–**e**) and (**q**). *CC* connecting cilium, *GC* ganglion cell layer, *INL* inner nuclear layer, *IPL* inner plexiform layer, *IS* inner segment of photoreceptors, *ONL* outer nuclear layer, *OPL* outer plexiform layer, *OS* outer segment of photoreceptors, *RPE* retinal pigmented epithelium.

To further evaluate the possible role of the *ALPK1* variant in this disease, expression levels of *ALPK1* were assessed via quantitative real-time PCR in human donor eye tissue as well as in FFPE spleen tissue from two affected individuals (family 1, II-3 and III-2) along with two age-matched controls. All donor eye tissue was processed at 6 hours or less postmortem time as previously described.^16^ In the eye, *ALPK1* expression was evaluated in the optic nerve, macula, RPE, and extramacular retina. Pooled RNA from two normal eyes was reverse transcribed and real-time PCR was performed. The resulting data demonstrates increased expression of *ALPK1* in the RPE and optic nerve compared with retina (Fig. 3a). Real-time PCR performed on spleen tissue samples confirmed the expression of *APLK1* in the normal spleen (data not shown). However, the quality of the RNA obtained from the FFPE tissue was too low to effectively compare diseased tissue samples with normal samples.

In the mouse retina, confocal microscopy showed that Alpk1 localized to the region of the photoreceptors where the basal bodies of connecting cilia are located and to the RPE, with some additional distribution across the inner and outer plexiform layers (Fig. 3b, c). In the connecting cilium region, costaining with a pancentrin antibody^17^ indicated localization of Alpk1 to the basal body of the photoreceptor cilium and adjacent centriole region (Fig. 3c–f). In mouse skin, Alpk1 was present in the sweat glands, especially in the myoepithelial cells where there was colocalization with the myoepithelial cell marker α-SMA (Fig. 3g–r). There was also broad expression in other mouse tissues (Supplementary Figure S1).

### ALPK1 subcellular localization in centrosomes, spindle poles, and primary cilia

**Fig. 4:**
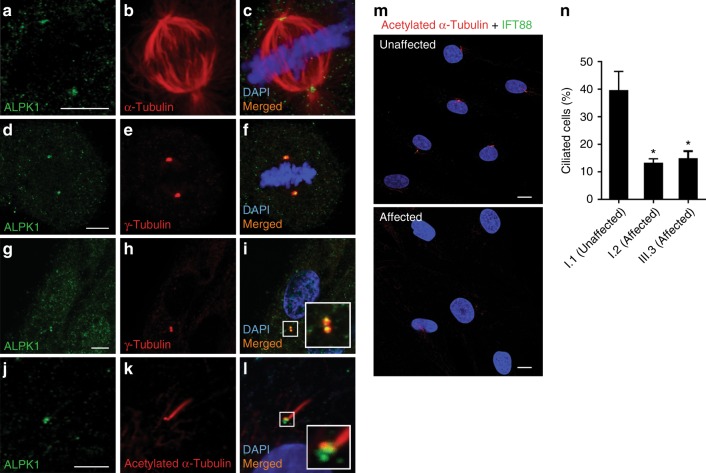
**ALPK1 localization to the centrosomes and base of the cilia in cells and the impact of ALPK1 (c.710C>T, [p.Thr237Met]) variant on primary cilia assembly.** (**a**–**c**) Antibody staining of fixed ARPE19 cells with anti-ALPK1 (green, Proteintech, cat #19107-1-AP) and anti-α-tubulin (red) antibodies in a mitotic cell shows the localization of ALPK1 in spindle poles. (**d**–**f**) Further immunostaining of ARPE19 cells with anti-γ-tubulin (red) confirmed the centrosomal localization of ALPK1 during mitosis. (**g**–**i**) Localization of ALPK1 in ARPE19 cells in interphase shows a diffuse cytosolic pattern and enrichment of ALPK1 in centrosomes of the cells, which colocalize with γ-tubulin. (**j**–**l**) ARPE19 cells were serum starved for 24 hours and immunostained with anti-ALPK1 (green, Proteintech, cat #19107-1-AP) and anti-acetylated-α-tubulin (red) showing the ALPK1 localization at the base of primary cilium. Cell nuclei are stained with DAPI (blue). (**m**) Fibroblast cells were treated with serum-free medium for 24 hours and labeled with primary cilia markers anti-acetylated-α-tubulin (red), anti-IFT88 (green), and DAPI (blue). Representative images of unaffected cells and affected cells carrying *ALPK1* (c.710C>T, [p.Thr237Met]) variant show decreased number of ciliated cells in fibroblasts from affected patients compared with control. (**n**) Quantification of the ciliated cells in one unaffected control and two affected patients. The analysis showed significant decrease in assembly of primary cilia in the affected patients carrying *ALPK1* (c.710C>T, [p.Thr237Met]) variant. The graphs show the means ± s.e.m. from three independent experiments where 100 cells were scored for each sample in each experiment. *p<0.05. Scale bars represent 5 μm in (**a**, **d**, **g**, **j**) and 10 μm in (**m**, **n**).

Our immunofluorescence studies on ARPE19 cells revealed the localization of ALPK1 in the spindle poles at metaphase (Fig. 4a–f), and in centrosomes of cells during interphase (Fig. 4g–i). Furthermore, cells were serum starved for 24 hours and then stained with ALPK1 antibody and acetylated α-tubulin, which is a marker for primary cilia. Super-resolution confocal imaging showed the presence of ALPK1 in the base of primary cilia (Fig. 4j–l). This suggested a possible role for ALPK1 in centrosome biology, which is critical for mitotic spindle pole assembly and primary ciliary assembly.^18^

### Abnormal cilia function due to *ALPK1* variant, p.Thr237Met

Given the centrosomal localization of ALPK1 and its presence in the basal regions of the primary cilium and the photoreceptor cilium, we hypothesized that the ALPK1 pathogenic variant, p.Thr237Met, may affect ciliogenesis. The evaluation of ciliogenesis in the Australian family revealed, on average, 40% of the cells were ciliated in an unaffected control, while only 15% and 13% of the cells could assemble the primary cilium in two affected individuals (*p*<0.05) (Fig. 4m, n). These findings suggest that phenotypic features in this family may relate to abnormalities of cilia–centrosomal function.^19^

### Increased proportion of multinucleated cells due to *ALPK1* variant, p.Thr237Met

In view of the presence of ALPK1 in the spindle poles of mitotic cells, we considered that the variant in this gene may affect cell division and cytokinesis. Transfection of HeLa cells with p.Thr237Met *ALPK1* compared with wild type, empty vector, and untransfected revealed that cells transfected with the mutant protein had a significantly higher number of multinucleated cells (16%), compared with the cells transfected with wild-type ALPK1 (3.7%) or empty vector (5.7%) (*p*<0.01) (Supplementary Figure S2).

## DISCUSSION

We define a phenotypically distinct autosomal dominant retinal dystrophy syndrome, ROSAH, with features of retinopathy, optic nerve edema, splenomegaly, anhidrosis, and headache in five unrelated families, and show heterozygous (c.710C>T, [p.Thr237Met]) *ALPK1* as the causative variant in all cases. Some patients also exhibit low-grade ocular inflammation and recurrent infections. Three independent exome and genome sequencing family studies led to the identification of this ALPK1 variant, which was confirmed in the two additional families. The occurrence of the same c.710C>T transition in a CG dimer in all five families indicates the existence of a mutational hotspot. ALPK1 is an ɑ-protein kinase of poorly defined function. Consistent with the phenotypic features in our patients, we showed that ALPK1 was highly expressed in retinal tissues, notably the retinal pigment epithelium and the connecting cilium region of photoreceptors, localized to myoepithelial cells of sweat glands, and was expressed across most other tissues. Our cellular investigations suggest a role for ALPK1 in centrosomal cilia function. Our findings show that ROSAH syndrome is a distinctive autosomal dominant retinal dystrophy syndrome, caused by a recurrent heterozygous variant in ALPK1, and indicate a role for ALPK1 across several different organ systems.

ALPK1 was first identified as a member of a family of atypical protein kinases, the ɑ-kinases, through homology to the catalytic domain of myosin heavy chain kinase.^20^ These kinases phosphorylate sites that are surrounded by peptide regions with ɑ-helical secondary structure.^21^ The ALPK1 catalytic domain lies between amino acids 1027 and the carboxy-terminus at 1244 (Fig. 1d).^22^ It has homology to the myosin heavy chain kinase of *Dictyostelium*.^20^ In addition, ALPK1 has been shown to phosphorylate myosin 1a in MDCK cells.^23^ In MDCK cells, ALPK1 has been shown to play a role in trafficking sucrose–isomaltase lipid rafts from the Golgi network through phosphorylation of myosin 1a.^23,24^ When disrupted, ALPK1 regulation of apical transport may affect cellular establishment of polarity.^24^

In all patients with ROSAH syndrome there is evidence of an early-onset retinal dystrophy. We noted presence of Alpk1 in the basal region of the connecting cilium of the photoreceptors (Fig. 3b–f), as well as the centrosomes and base of the primary cilium (Fig. 4a–l). We identified reduced ciliation of fibroblast cells from patients affected with ROSAH syndrome compared with controls. Pathogenic variants in centrosome–cilia proteins have been extensively described in syndromic and nonsyndromic forms of retinal dystrophy disease, such as pathogenic variants in *TOPORS*, *RPGR*, *RP2*, *CC2D2A*, and *CEP290* (refs. ^25–30^). These pathogenic variants indicate that ciliary–centrosomal abnormality is one of the known pathways that contributes to the pathogenesis of retinal dystrophy through defects in ciliary formation and regulation of cargo trafficking in the photoreceptors.^19^ In addition, cilia formation is linked with cell cycle progression^31^ and localization of ALPK1 at the spindle poles during mitosis (Fig. 4a–f) raises the possibility that abnormality of ALPK1 may impact cell cycle progression, another factor that may contribute to a retinal degeneration phenotype.^32^ Noting that the population database gnomAD did not reveal likely loss-of-function intolerance of *ALPK1*, we investigated the impact of overexpression of *ALPK1* (c.710C>T,[p.Thr237Met]) in HeLa cells and showed increased multinucleated cells (Supplementary Figure S2), suggesting a possible gain-of-function disease mechanism. ALPK1 was also expressed in the RPE where its role in maintenance of epithelial cell polarity may also be important for normal function of the RPE and prevention of retinal dysfunction. Patients with ROSAH syndrome also exhibited anhidrosis, so the role of ALPK1 in maintenance of epithelial cell polarity may also be relevant for normal function of the sweat glands.

There are several reports of proposed association of single-nucleotide polymorphisms (SNPs) in *ALPK1* with increased susceptibility to inflammatory conditions, including gout and chronic kidney disease.^33,34^ In the mouse, Alpk1 was found to mediate innate immune responses to bacterial infection^35^ and Alpk1 abnormality was found to be associated with increased susceptibility to inflammatory bowel disease.^36^ The role of ALPK1 in inflammation may be mediated through regulation of NF-κB signaling.^37^ Interestingly, the N terminal region of ALPK1, including the Thr237 amino acid affected in our patients, has been found to be critical for stimulation of the ALPK1 kinase domain and phosphorylation and activation of downstream targets in the NF-κB signaling pathway.^35^ Patients with ROSAH syndrome also show signs of inflammation, exhibiting recurrent or chronic low-grade uveitis, although the uveitis does not appear to be clinically alleviated by immunosuppression. Abnormalities of the innate immune pathway may also contribute to retinal degeneration, raising a further avenue for investigation in ROSAH syndrome patients. Some patients with ROSAH syndrome also exhibit episodic infections, fevers, and pancytopenia, further indicating an inflammatory component to this condition.

Our report is the first where a pathogenic variant in *ALPK1* is found in affected individuals with Mendelian disease. Motor coordination deficits of unknown mechanism have been found in mice with the catalytic domain of ALPK1 disrupted by a piggyBAC transposon.^38^ None of the individuals in the families we describe have motor coordination defects. These mice and the Alpk1 loss-of-function mice with inflammatory abnormalities were not tested for blindness or retinal dystrophy.^35,36^ The etiology of the optic nerve edema, splenomegaly, and headache is unclear in ROSAH syndrome patients. Two patients had lumbar punctures performed due to concerns about raised intraocular pressure because of the appearance of the optic nerve head, but no increased pressure was detected. ALPK1 may be important in the development of the red blood cell (RBC) cytoskeleton, which if impaired may lead to the propensity to form elliptocytes noted in one case, and may be filtered at the level of the spleen resulting in splenomegaly. In addition, our patients exhibit headache, which has been associated with alterations in kinase function affecting calcitonin gene related peptide and trigeminal nerve pain.^39,40^

In summary, we name this autosomal dominant condition ROSAH syndrome to reflect the most common features across affected individuals: retinopathy, optic nerve edema, splenomegaly, anhidrosis, and headache, and identify p.Thr237Met *ALPK1* as the causative variant. Future studies are warranted to examine the precise pathophysiological mechanism of this new autosomal dominant retinal dystrophy syndrome.

## URLs

1000 Genomes, http://www.internationalgenome.org/; dbSNP, http://www.ncbi.nlm.nih.gov/SNP/; ExAC Browser, http://exac.broadinstitute.org/; Integrative Genomics Viewer (IGV), http://www.broadinstitute.org/igv/; MutationTaster, http://www.mutationtaster.org/; NCBI Genome, http://www.ncbi.nlm.nih.gov/genome/; NCBI Nucleotide, http://www.ncbi.nlm.nih.gov/nuccore/; NCBI Primer designing tool, https://www.ncbi.nlm.nih.gov/tools/primer-blast/; NHLBI Exome Sequencing Project, http://evs.gs.washington.edu/EVS/; OMIM, http://www.omim.org/; Picard tools, http://picard.sourceforge.net/; PolyPhen-2, http://genetics.bwh.harvard.edu/pph2/; SIFT, http://sift.bii.a-star.edu.sg/; UCSC Genome Browser, http://genome.ucsc.edu/; UniProt, http://www.uniprot.org/.

## Supplementary information

Supplementary Information
