# Supplementary material for: ALPK1 missense pathogenic variant in five families leads to ROSAH syndrome, an ocular multisystem autosomal dominant disorder
Source: Genet Med. 2019 Apr 10;21(9):2103–15. doi: 10.1038/s41436-019-0476-3 (PMC6752478; doi:10.1038/s41436-019-0476-3)
Supplement: Supplementary file 1 — Supplementary Information [file 41436_2019_476_MOESM1_ESM.docx]

**Supplementary**

**(Williams, Javed, Sabri, Morgan et al)**

**Supplementary Methods: Clinical Examination Locations**

All members of the Utah cohort were examined at the University of Utah. All members of the Australian cohort were examined at The Children’s Hospital at Westmead, Sydney, and the Save Sight Institute, University of Sydney. The individuals from the Netherlands family were examined at the Erasmus MC at Rotterdam, The Netherlands, and The Rotterdam Eye Hospital at Rotterdam, The Netherlands. Members of the Delaware cohort were examined at Wills Eye Institute and at the National Eye Institute / National Institutes of Health. Members of the Virginia family were examined at the clinic of Dr. Farley, Polo and Ho in Colonial Heights, Virginia. The methods and results of these examinations for the Utah cohort are described in Tantravahi *et al*^1^ and herein for newly phenotyped individuals from the Utah cohort, and members of the Australian, Netherlands, Virginia, and Delaware families.

**Supplementary Methods: Exome or Genome Sequencing and Alignment, Families 1,2 and 3**

*Exome sequencing - Family 1 (Utah)*

Exonic DNA was captured using previously described techniques (Illumina TruSeq Exome Enrichment Kit). 101 Cycle Paired End Sequencing was conducted with the Illumina HiSeq2000, per the manufacturer’s protocol. All data generated from the Illumina platform was mapped and aligned to hg19 using BWA. The data was processed using Picard tools to identify and discard duplicate reads. The UnifiedGenotyper in the Genome Analysis Tool Kit (GATK) was used to identify single nucleotide polymorphisms (SNPs) and indels.^2; 3^ For manual inspection and curation of alignments, Integrative Genomics Viewer (IGV) was used. For all WES subjects, the overall average sequencing coverage was 40X.

*Genome sequencing – Family 2 (Australia)*

DNA samples for genome sequencing were covaris-sheared according to the manufacturer's recommendation for 300bp. End-repairing, 3' A-tailing and ligation of Illumina adapter (Illumina's Index PE Adapter Oligo Mix, #1005711) were done using NEBNext DNA Library Prep Master Mix Set for Illumina (#E6040L). Samples were enriched using Illumina's PCR Primer InPE 1.0, 2.0 and a mix of PCR Primer Index oligos (#1005712-25) with 6 PCR cycles. Sequencing of libraries was done using Illumina's HiSeq 2000 2X101bp run with 4 lanes of sequencing per sample. The overall average sequencing coverage for each sample was 44X. The recommended best practice pipeline for GATK (v 2.8.1) was used to process the raw sequencing data.

*Exome sequencing – Family 3 (Netherlands)*

Exome capture was performed with the Agilent SureSelect Clinical Research Exome (CRE) Capture enrichment kit (Agilent Technologies). Exome sequencing was performed on the Illumina HiSeq platform (BGI, Copenhagen, Denmark). Reads were mapped to the genome hg19 with BWA. Variants were called with the GATK.

**Supplementary Method: Cell lines**

Human fibroblasts were established from affected and unaffected members of Family 2 (Australia) and checked for the *ALPK1* variant (c.710C>T, [p.Thr237Met]) by Sanger sequencing. The ARPE-19 cell line was a gift from Dr. Michele Madigan (Save Sight Institute, Sydney, Australia) and the HeLa cell line was a gift from Dr. Megan Chircop (Cell Cycle Unit, Children’s Medical Research Institute, Sydney, Australia). Cell line authentication was not carried out by the authors. Mycoplasma testing was undertaken annually for all cells and showed negative results.

**Supplementary Clinical Information**

Family 1 (Utah, US)

The phenotype of ROSAH syndrome in the Utah cohort was previously reported in Tantravahi et al,^3^ and now additional members of the Utah cohort, have been examined in the ophthalmology clinics at the University of Utah. Individual’s I-1 and I-2, the parents of affected Patient II-3 (Family 1, Figure 1A), were found to have normal visual acuities and ophthalmic exams and did not exhibit any signs of ROSAH syndrome. Four grandchildren of Patient II-3 have also been identified. Individual IV-3 has not been available for exam or genotyping. Individuals IV-1 and IV -2 were found to have normal visual acuity and ophthalmic exams. These individuals also did not have the disease-causing mutation (Figure 1A). Patient IV-4, who was heterozygous for the disease-causing mutation, had a clinical course complicated by alpha-1-antitrypsin deficiency leading to end-stage liver disease, for which she has required liver transplantation. Her ophthalmic exam, at age 2 years, by a pediatric ophthalmologist has not revealed any abnormalities.

Family 2 (Australia)

Six members of a three generation Australian family (Family 2, Figure 1A, Table 1) are described here. The proband in the Australian cohort, Patient III.2, a 9-year-old male (Figures 1A, 2A-D), presented for genetics review after a vitreous hemorrhage on a background of optic nerve abnormality and retinal dystrophy and a family history of similar features. Patient III.2 started experiencing reduced vision from the age of 7 years. Following resolution of the hemorrhage, his best spectacle corrected visual acuity (BSCVA) was 20/250 in the right eye and 20/200-1 in the left eye. He had reduced color vision. Retinal assessment revealed no obvious peripheral retinoschisis, telangiectactic vessels, or retinal neovascularization. There was disturbance of both maculae with loss of foveal reflexes. Optic discs had edema (Figure 2A). There was mild peripheral retinal atrophy and no pigmentary retinal changes. Fundus autofluorescence showed generalized hyperautofluorescence extending around the posterior pole anterior to the vascular arcades (Figure 2D), and OCT showed retinal and peripapillary generalized thickening with indistinct retinal layers (Figure 2E), compared with control (Figure 2G). There were cystic changes in the macula and loss of the photoreceptor inner segment and outer segment reflectivity. Full field electroretinography (ERG) showed reduced scotopic and photopic responses, with the photopic more severely affected (Figure 2F compared with control (Figure 2H). Pattern ERG measuring macula function was almost extinguished in both eyes for the central 15 degrees. In view of the optic disc edema, a neurological assessment, lumbar puncture, and MRI brain were performed, all of which were normal. He also reported anhidrosis and intermittent migraine headaches, but was an otherwise well grown and active child with outstanding ability in gymnastics. Laboratory investigations including full blood count (FBC) and coagulation screen were normal apart from a mildly elevated urea.

In the Australian cohort, the proband’s mother (Patient II.3, Family 2, Figure 1A), aged 38 years, experienced visual impairment from 5 years of age with gradual deterioration becoming more noticeable by the age of 21 years. Optic nerve changes were reported to have been present early in her disease process. At age 38 years, she had partial light perception in each eye with sensory-deprivation exotropia and bilateral posterior subcapsular cataracts. OCT showed thickening of the retina (Figure 2I) and fundoscopy showed patchy pigmentation, with a partial bony spicule pattern (Figure 2J, K). Only the left optic disc could be viewed and it showed disc pallor with attenuated arterioles. There was no disc edema and there was mild lens subluxation. Patient II.3 also reported that in the preceding year, at age 37 years, she had viral meningitis following varicella zoster exposure. At that time she also developed generalized cytopenia for which she had required multiple blood transfusions. Since this episode, she has been found to have mild renal impairment. She also reported frequent and recurrent headaches over the years, fevers and anhidrosis. Laboratory investigations, were all normal, apart from mildly elevated urea and creatinine.

The proband’s maternal grandmother, (Patient I.2, Family 2, Figure 1A), at age 65 years, had no light perception bilaterally. Her vision impairment was from her late childhood years, and she had been told she had an optic nerve and retinal abnormality and developed sensory-deprivation exotropia. Fundoscopy showed extensive and dense pigmentary changes throughout the retina. She had dense nuclear sclerotic cataracts with crystalline lens subluxation inferonasally. The fundus could not be clearly viewed secondary to dense cataracts. She reported anhidrosis, and had been otherwise well. Laboratory investigations were all normal, apart from mildly elevated urea and creatinine.

The proband’s maternal uncle, (Patient II.2, Family 2, Figure 1A), at age 35 years, also had no light perception, with vision impairment noted from mid childhood, and similar ophthalmic features to his mother, Patient I.2, and sister, Patient II.3. He had splenomegaly in his late teenage years, and a splenectomy was performed at 20 years of age for “hereditary spherocytosis”, although no spherocytes were noted on blood examination. He also had anhidrosis. Laboratory investigations were all normal.

The proband’s older sister, (Patient III.1, Family 2, Figure 1A), at age 12 years did not have any complaints with near, distance or night vision. Her BSCVA was 20/50-2 in the right eye and 20/32 in the left eye. She had moderate hypermetropia. Fundoscopy showed subtle disc edema on both sides with blurred disc margins. There was subtle macula disturbance on the right side and a normal appearing left macula and an intact foveal reflex. There was mild attenuation of the retinal arterioles. There were no abnormal peripheral pigmentary changes. OCT showed thickening and cystic changes in the retina. ERG showed a cone-rod pattern of dysfunction. She also experienced anhidrosis. Later that year, at the time of an upper respiratory infection, she was found to have splenomegaly (6-7cm below the L costal margin) and petechiae in her mouth. Laboratory investigations were all normal, apart from mildly elevated urea.

The proband’s younger brother, (Patient III.3, Family 2, Figure 1A), at age 7 years was experiencing difficulty with schoolwork and reading but had no difficulties with night vision. His uncorrected visual acuity was 20/50-2 in the right eye and 20/63-1 and he was slightly hypermetropic. Fundoscopy showed elevation of the optic discs with blurred disc margins. Peripapillary splinter hemorrhage were noted superonasally in the right eye. The maculae had early loss of foveal reflexes. There were no abnormal pigmentary changes or any arteriolar attenuation. OCT showed thickening and cystic changes in the retina. ERG subsequently showed cone and rod dysfunction. He was noted to have splenomegaly (3-4cm below the costal margin) and also experienced anhidrosis. Laboratory investigations, were all normal, apart from mildly elevated urea.

Other members of the Australian cohort, including I.1, II.1 and II.4, did not have any of the abnormal ocular or systemic features that were seen in the affected family members. Histopathology and electron microscopy studies on skin from Patient II.3, in the Australian cohort, did not reveal any overt pathological features. Histopathology on spleen from Patient II.2, in the Australian cohort, revealed some congestion, but no marked distinguishing features.

Family 3 (Netherlands)

The proband in the Netherlands family (Patient II.3, Family 3, Figure 1A) is a 34-year-old white male born to non-consanguineous parents. At the age of 7 years, he started experiencing reduced vision while his BSCVA was 20/25 in both eyes. Fundoscopy showed bilateral swollen optic disks. Neurological examination did not show intracranial disease. Serological and lumbar puncture- examination did not show infections or other known optic neuritis inflammatory diseases. No agent to cause optic nerve toxicity was found. Besides a hereditary plasminogen deficiency which is also present in other family members, no etiology or other systemic disease was confirmed during extensive screening. Recurrent ophthalmologic examination showed diffuse retinal vascular leakage, with vitreous proteins, macular edema and swollen optic disks. Over the next years, recurrent episodes of vitritis and macular edema developed. The inflammation was extensively treated with oral steroids, cyclosporin-A and adalimumab, and macular edema with acetazolamide, octreotide, triamcinolone injections and ozurdex over the years. A vitrectomy was performed in the right eye at the age of 11 years. Cataract surgery was performed at the age of 17 and 21 years respectively in the right and left eyes. Despite all these therapeutic modalities, the optic nerves gradually evolved to complete optic atrophy. At the age of 26 years, BSCVA was no light perception OD and hand movements OS, and currently BSCVA OS has deteriorated to only light perception.

His further history revealed broadening of his joints with complaints of stiffness existing since his childhood. Furthermore, he developed progressive halluces valgi with an unusual early-onset in childhood. He suffers from anhidrosis and has notably dry skin. In his childhood, he had periods with headaches but these attacks disappeared in his teenage years. Laboratory investigations showed a chronic mild microcytic anemia without a known cause. Occasional mild thrombocytopenia and lymphopenia was observed during viral or other infections. No splenomegaly with physical or ultrasound examination was present. An unexplained elevated angiotensin converting enzyme was detected in his early twenties without signs of sarcoidosis or granulomatosis disease.

Genetic investigation with copy number analysis using SNP array showed a normal male profile.

His parents and three brothers reported none of the ocular or systemic features suggestive of ROSAH syndrome.

Family 4 (Virginia, US)

Patient II-3 was diagnosed with chronic anemia at age 11 years. She first noticed decreased vision in her early teens and was diagnosed with angle closure glaucoma by age 28. At age 37 optic disc edema was diagnosed in her right eye. By the age 42, vision was 20/50 in right eye, however, the left eye had decreased to 20/200. IOP was noted as 10mmHg in the right eye and 40 mmHg in the left. At age 46, the patient noticed an abrupt change in vision in the right eye; visual acuity was 20/400. Fluorescence angiography (FA) suggested optic disc edema in both eyes with associated macular edema OD. By age 52 vision was 20/400 in her right eye and light perception with projection in the left. The patient's most recent examination at age 57 revealed visual acuity at 20/400 and NLP OS. Exam findings included bilateral optic nerve head edema, macular degeneration and dense nuclear sclerotic cataracts.

Patient III-2 was diagnosed with papilledema at age 7 years. Visual field studies demonstrated a large cecocentral scotoma on both the right and left fields. At age 9 years the patient’s signs and symptoms included anhidrosis, abnormal peg-shaped teeth, excessive number of cavities, increased susceptibility to upper respiratory infections, frequent nose bleeds, dry mouth and headaches exacerbated by heat. A genetic consult led to a diagnosis of autosomal ectodermal dysplasia. By age 11 years, her vision had decreased to 20/200 in both eyes. Patient III-2 had a subjective and objective improvement in vision at age 16. Visual acuity was measured at 20/80 right eye and 20/100 left eye. The patient’s most recent exam at age 30, revealed stable papilledema and an unspecified retinal dystrophy. The vision decreased to 20/400 in both eyes with significant mobility problems

Patient IV-1 was diagnosed with papilledema at age 9 years. At the time of diagnosis her visual acuity was 20/20 in both eyes. At her most recent examination, at age 11 years, the papilledema persisted and her visual acuity was 20/25 in both eyes.

Family 5 (Delaware, US)

Patient II-1 is currently an 18 year old, white male. He was healthy till the age of 8 years when he had a febrile streptococcal infection that did not resolve despite treatment with Augmentin. He had persistent fever and became jaundiced. On hospital admission splenomegaly and pancytopenia was discovered. He was clinically icteric with indirect bilirubin of 7.1mg/dL total bilirubin 8.2mg/dL, anemic (8.5 g/dL of Hemoglobin), thrombocytopenic (105,000 platelets/ml) and leukopenic (2,400 cells/ml). Peripheral smear showed elliptocytes and spherocytes. He was evaluated with a marrow aspirate that aside from increased red cell precursors was normal. EBV and CMV PCR were negative. No granulomatous disease and no lymphoma were found. His illness resolved without a definitive diagnosis. Subsequently he developed cyclical recurrences of fever, jaundice, elevated indirect bilirubin, malaise, thoracic level back pain and exacerbation of cytopenias that lasted 24-48 hours every 4-6 weeks with spontaneous recovery back to normal each time. He does not suffer from migraine headaches.

Over the following years, splenomegaly persisted. He was diagnosed with Gilbert syndrome. At age 10 years, repeat bone marrow evaluation was again normal. Also, during that year, bilateral intermediate uveitis and mild optic disc edema were found on routine ophthalmologic examination. The uveitis was treated using periocular steroid injection. At age 11 years, hemoglobin electrophoresis, red cell enzyme assessment and osmotic fragility testing were conducted and all were normal.

At age 13 years, he had a particularly severe illness with fever and jaundice lasting about 3 days. Eye evaluation at this time was significant for nyctalopia with new evidence of an unidentified retinal photoreceptor dystrophy. Ocular examination revealed optic nerve edema (Figure 2L), and OCT showed thickening of the retina and indistinct retinal layers (Figure 2M). Goldmann visual fields demonstrated significant constriction of his visual field (Figure 2N). ERG showed rod greater than cone dysfunction.

An extensive evaluation of immune function was normal aside from a mildly low IgM and low mitogen stimulation to pokeweed. Liver transaminases were mildly elevated. A liver biopsy showed thickened hepatic arterioles, focal periarterial intimal fibrosis in some hepatic arterioles, also mild hemorrhage and congestion around several central veins with a mild lymphohistiocytic infiltrate all suggesting an imbalance between portal venous and hepatic arterial blood flow. Concavalin and phytohemagglutinin levels were normal. Angiotensin converting enzyme was normal, genetic assessment with a periodic fever panel (Genzyme) was normal. Cytokines including IL-1β, TNF-α and IL-6 were unrevealing. Flow cytometry for hereditary spherocytosis was also negative. Celiac evaluation was negative for celiac disease. There was no evidence of malabsorption. Vitamins A, E, K, B12, and folate were normal. Genetic testing for autoimmune lymphoproliferative diseases and storage diseases, including Gaucher’s Disease were negative. Assessment for paroxysmal nocturnal hemoglobinuria and an extensive assessment for autoimmune diseases were negative. G6PD testing was negative. An MRI of the spine showed findings compatible with increased hematopoiesis in vertebral bodies and extraspinal soft tissues at T5-6 possibly explaining his back pain during exacerbations. Repeat peripheral smears were notable for elliptocytosis.

The affected individual continues to have episodic periods of fevers, fatigue and back pain every 4-6 weeks that last for 24-48 hours and resolve. His most recent ocular examination at age 17 years showed Snellen BSCVA of 20/160 in each eye with significant posterior subcapsular cataracts. His uveitis was quiet with zero grade cells in the anterior chamber and +3 old pigmented vitreous cells with no vitreous haze in either eye. Recurrences of ocular inflammation between age 15 through 17 were controlled with the use of intraocular corticosteroids and a course of Adalimumab. He also received Rituximab and IVIG for systemic symptoms which may have contributed to the control of his uveitis. He failed a D15-Farnsworth color vision test with a deficit along the tritan axis. Retinal exam showed peripheral hypopigmentation with atrophy and moderately attenuated vessels. Scotopic and photopic ERG were at noise level. The optic nerve head was elevated but pale in color. Macular OCT showed loss of ellipsoid zone OU with no frank macular edema and fluorescein angiogram did not reveal any retinal vascular leakage.

| **Suppl. Table S1.** List of primary and secondary antibodies used in immunofluorescence studies | | | |
| --- | --- | --- | --- |
| Antibody name | Company, Catalogue number | Dilution |  |
| *Primary antibodies* |  |  |  |
| Rabbit anti-ALPK1 | Proteintech, cat# 19107-1-AP | 1:200 |  |
| Mouse anti-α-tubulin | Sigma-Aldrich, cat# T9026 | 1:1000 |  |
| Mouse anti-acetylated-α-tubulin | Sigma-Aldrich, cat# T6793 | 1:500 |  |
| Mouse anti-γ-tubulin | Sigma-Aldrich, cat# T5326 | 1:200 |  |
| Rabbit anti-IFT88 | Proteintech, cat# 13967-1-AP | 1:1000 |  |
| Rabbit polyclonal anti-ALPK1 | MyBioSource, cat# MBS001969 | 1:100 |  |
| Mouse monoclonal anti-centrin | Millipore, cat# 04-1624 | 1:600 |  |
| Mouse monoclonal anti α-smooth muscle actin | Sigma Aldrich, cat# A2547 | 1:100 |  |
|  |  |  |  |
| *Secondary antibodies* |  |  |  |
| Donkey anti-rabbit Alexa Fluor 488 | Life Technologies, cat# R37118 | 1:1000 |  |
| Donkey anti-mouse Alexa Fluor 594 | Life Technologies, cat# A-21203 | 1:1000 |  |
| Donkey anti-rabbit Alexa Fluor 647 | Life Technologies, cat# A-31573 | 1:1000 |  |
|  |  |  |  |
| *Nucleus staining* |  |  |  |
| 4', 6-Diamidino-2-Phenylindole, Dihydrochloride (DAPI) | Sigma-Aldrich, cat# D9542 | 1:1000 |  |

**Suppl. Figure S1.** Alpk1 mRNA expression in the mouse tissues

RNA was isolated from mouse (Arc(S)) retina at day 5 and day 10, optic nerve, adult retina, eye, brain, heart, lung, stomach, liver, spleen, intestine, and kidney, reverse transcribed and amplified by PCR with a mouse ALPK1 specific primer pair. Analysis of amplification products by agarose gel demonstrated broad expression of ALPK1 in mouse tissues. β-actin was used as a control.


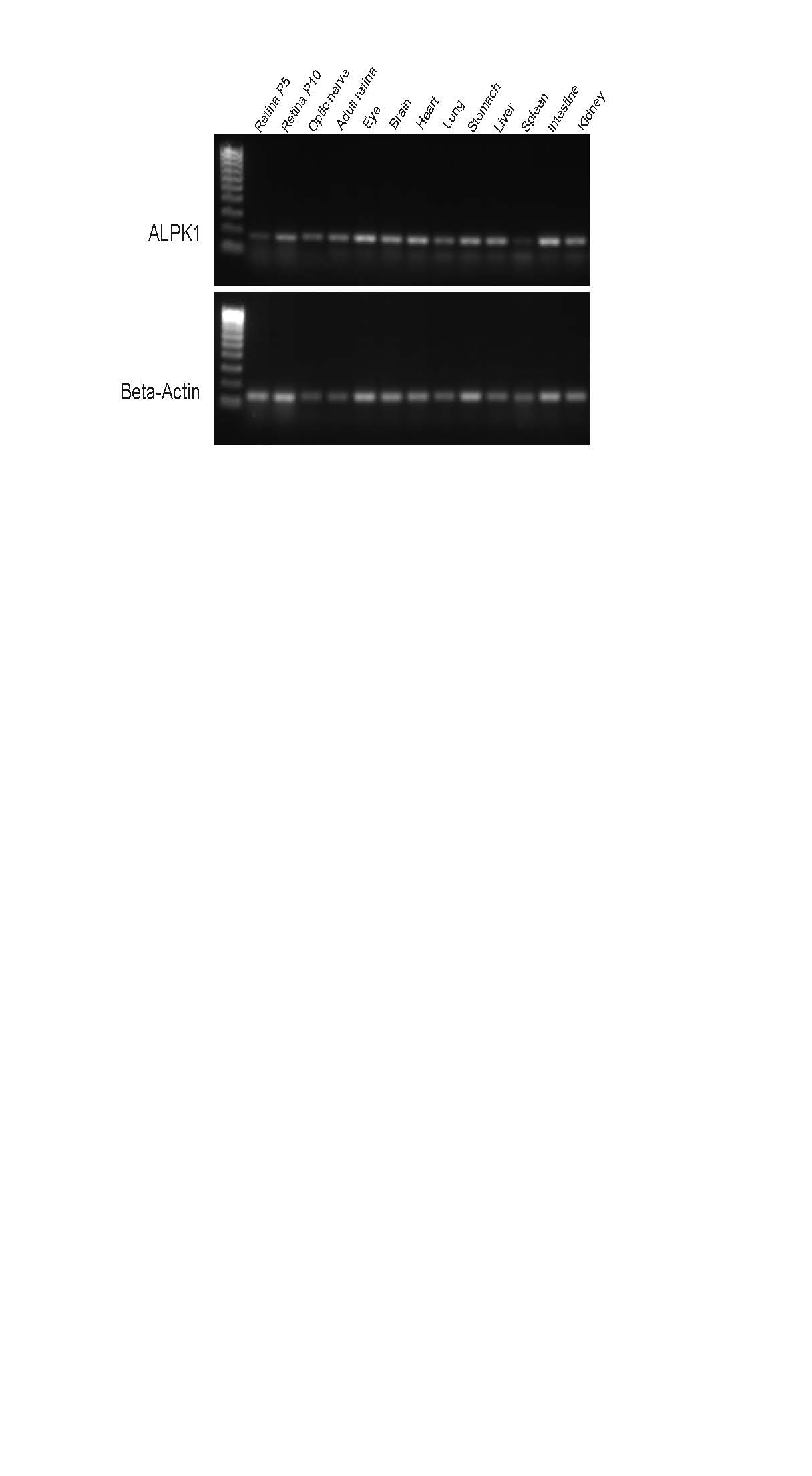


**Suppl. Figure S2.** Transfection of HeLa cells with ALPK1 constructs and multinucleation assessment.

HeLa cells were transfected with GFP-ALPK1 (WT), GFP-ALPK1 (c.710C>T, [p.Thr237Met]), or GFP-only vector (empty vector). Untransfected HeLa cells were used as a control. Cells were stained with α-tubulin and DAPI and analyzed for the number of multinucleated cells 48 hours post-transfection. Quantification analysis (as per Chircop *et al*^4^) under the four different conditions showed significant increase of multinucleated cells after GFP-ALPK1 (c.710C>T, [p.Thr237Met]) transfection while wild type ALPK1 did not show a significant increase in multinucleation compared with untransfected cells. The graphs show the means ± s.e.m. from three independent experiments where 100 cells were scored for each sample in each experiment. ns, not significant; ***p*<0.01.


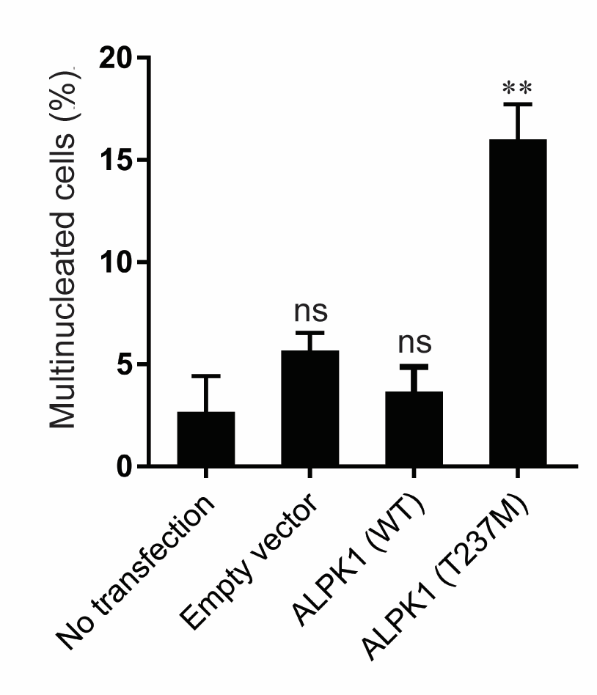


1. Tantravahi, S.K., Williams, L.B., Digre, K.B., Creel, D.J., Smock, K.J., DeAngelis, M.M., Clayton, F.C., Vitale, A.T., and Rodgers, G.M. (2012). An inherited disorder with splenomegaly, cytopenias, and vision loss. Am. J. Med. Genet. A. 158A, 475-481.

2. DePristo, M.A., Banks, E., Poplin, R., Garimella, K.V., Maguire, J.R., Hartl, C., Philippakis, A.A., del Angel, G., Rivas, M.A., Hanna, M., et al. (2011). A framework for variation discovery and genotyping using next-generation DNA sequencing data. Nature genetics 43, 491-498.

3. McKenna, A., Hanna, M., Banks, E., Sivachenko, A., Cibulskis, K., Kernytsky, A., Garimella, K., Altshuler, D., Gabriel, S., Daly, M., et al. (2010). The Genome Analysis Toolkit: a MapReduce framework for analyzing next-generation DNA sequencing data. Genome Res. 20, 1297-1303.

4. Chircop, M., Perera, S., Mariana, A., Lau, H., Ma, M.P., Gilbert, J., Jones, N.C., Gordon, C.P., Young, K.A., Morokoff, A., et al. (2011). Inhibition of dynamin by dynole 34-2 induces cell death following cytokinesis failure in cancer cells. Molecular cancer therapeutics 10, 1553-1562.
